# Core Metabolism Shifts during Growth on Methanol versus Methane in the Methanotroph *Methylomicrobium buryatense* 5GB1

**DOI:** 10.1128/mBio.00406-19

**Published:** 2019-04-09

**Authors:** Yanfen Fu, Lian He, Jennifer Reeve, David A. C. Beck, Mary E. Lidstrom

**Affiliations:** a Department of Chemical Engineering, University of Washington, Seattle, Washington, USA; b Department of Microbiology, University of Washington, Seattle, Washington, USA; c eScience Institute, University of Washington, Seattle, Washington, USA; Harvard University; University of East Anglia; Auburn University

**Keywords:** 13C tracer analysis, flux balance analysis, methanol, methanotrophs

## Abstract

Methylomicrobium buryatense 5GB1 is an obligate methylotroph which grows on methane or methanol with similar growth rates. It has long been assumed that the core metabolic pathways must be similar on the two substrates, but recent studies of methane metabolism in this bacterium suggest that growth on methanol might have significant differences from growth on methane. In this study, both a targeted metabolomics approach and a ^13^C tracer approach were taken to understand core carbon metabolism in M. buryatense 5GB1 during growth on methanol and to determine whether such differences occur. Our results suggest a systematic shift of active core metabolism in which increased flux occurred through both the Entner-Doudoroff (ED) pathway and the partial serine cycle, while the tricarboxylic acid (TCA) cycle was incomplete, in contrast to growth on methane. Using the experimental results as constraints, we applied flux balance analysis to determine the metabolic flux phenotype of M. buryatense 5GB1 growing on methanol, and the results are consistent with predictions based on ATP and NADH changes. Transcriptomics analysis suggested that the changes in fluxes and metabolite levels represented results of posttranscriptional regulation. The combination of flux balance analysis of the genome-scale model and the flux ratio from ^13^C data changed the solution space for a better prediction of cell behavior and demonstrated the significant differences in physiology between growth on methane and growth on methanol.

## Abstract (precis)

One-carbon compounds such as methane and methanol are of increasing interest as sustainable substrates for biological production of fuels and industrial chemicals. The bacteria that carry out these conversions have been studied for many decades, but gaps exist in our knowledge of their metabolic pathways. One such gap is the difference between growth on methane and growth on methanol. Understanding such metabolism is important, since each has advantages and disadvantages as a feedstock for production of chemicals and fuels. The significance of our research is in the demonstration that the metabolic network is substantially altered in each case and in the delineation of these changes. The resulting new insights into the core metabolism of this bacterium now provide an improved basis for future strain design.

## INTRODUCTION

Methylomicrobium buryatense 5GB1 is an obligate type I methanotroph and is a candidate with promise for converting methane into valuable chemicals in industrial processes (1–3). Recently, much progress has been made toward a basic understanding of M. buryatense 5GB1 metabolism during growth on methane (4–7). This obligate methylotroph is also able to grow on methanol with a growth rate similar to that seen with methane (4). Similarly to methane-based biotechnology, methanol-based biotechnology is a rapidly moving area (8, 9). Having a single strain that could use either methane or methanol creates more flexibility for future bioprocesses than is provided by strains that grow on only one of these one-carbon substrates.

**FIG 1:**
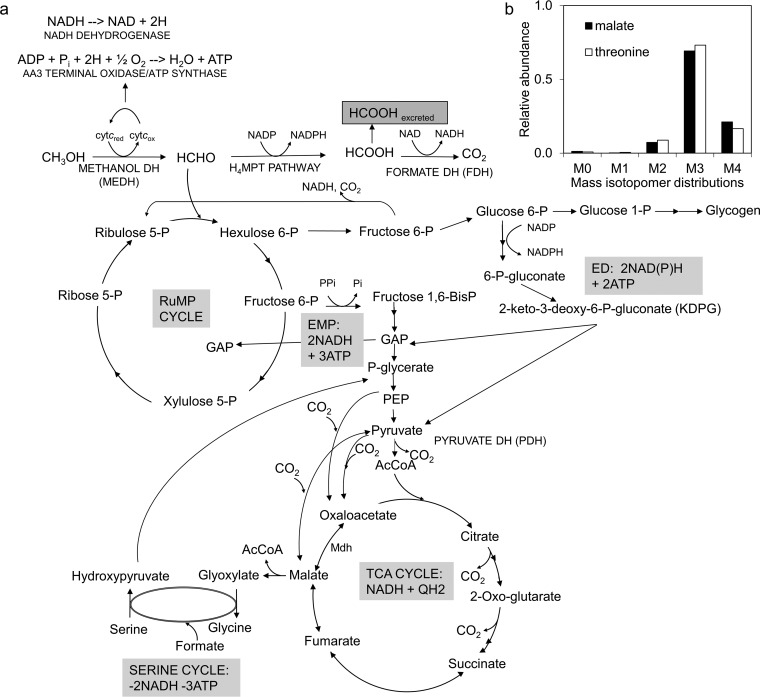
Core metabolism for growth on methanol with MIDs for key metabolites. (a) Methanol is oxidized to formaldehyde, which either enters into the RuMP cycle or is further oxidized to CO_2_. Sugar phosphates with a six-carbon (C6) backbone are then converted into sugar phosphates with a three-carbon backbone via either the EMP pathway or the ED pathway. Glycogen is synthesized from C6 sugar phosphates. Downstream of the PEP and pyruvate node, multiple routes exist for the interconversion of C3-C4 compounds. Both pyruvate dehydrogenase and malyl-CoA lyase could contribute to *de novo* AcCoA synthesis. AcCoA enters the TCA cycle to provide precursors for biomass synthesis. (b) MIDs of malate and threonine indicate negligible flux through fumarase to malate (see Table S2 in the supplemental material).

It might be expected that growth on methane and growth on methanol would use similar metabolic pathways, since the steps after methanol are the same (Fig. 1). In this scenario, methanol dehydrogenase converts methanol into formaldehyde, which can further be oxidized into formate and then CO_2_ to provide reducing equivalents as NADH. A portion of the formaldehyde is assimilated via the ribulose monophosphate (RuMP) cycle. Two variants of the RuMP cycle are predicted, namely, the Embden-Meyerhof-Parnas (EMP) variant and the Entner-Doudoroff (ED) variant (5). In the EMP pathway, fructose-6-phosphate (F6P) is converted into fructose 1,6-bisphosphate (FBP) and split into C3 sugar phosphates via fructose-bisphosphate aldolase, eventually resulting in pyruvate formation. In the ED pathway, 6-phosphogluconate (6PG) is converted into 2-keto-3-deoxy-6-phosphogluconate (KDPG) and then to pyruvate and glyceraldehyde 3-phosphate (GAP) via 2-keto-3-deoxygluconate 6-phosphate aldolase.

An analysis of growth on methane by this methanotroph has predicted that the electrons driving methane oxidation are mainly derived from methanol oxidation, with only a small proportion derived from NADH (4, 5). If that is the case, it raises the possibility that growth on methane and growth on methanol might be significantly different, since during growth on methanol, the electrons from methanol oxidation must enter the electron transport chain, consuming O_2_ and pumping protons, resulting in ATP synthesis (Fig. 1). Thus, growth on methanol should result in less NADH dehydrogenase flux and less ATP made through oxidative phosphorylation from NADH than growth on methane. That in turn could impact flux through other parts of central metabolism to rebalance levels of ATP and NADH production and consumption. It has already been observed that the levels of both excreted formate and intracellular glycogen are about 20-fold higher in methanol-grown cultures than in methane-grown cultures (4). In order to explore whether further metabolic differences exist, we compared the metabolism of M. buryatense 5GB1 grown on methanol to that already determined during growth on methane.

Metabolite profiling and ^13^C tracer analysis are two approaches that provide a direct view of cellular behavior resulting from an integrative effect on gene expression and regulation. In this study, targeted metabolite profiling was performed for core metabolites in cultures grown on either methane or methanol to discover and quantitate which parts of the network change when either substrate is utilized. In addition, ^13^C tracer analysis was conducted to determine whether the organism changes strategies for interconversion of C2-C3-C4 compounds downstream of the pyruvate node, based on growth substrate.

The data from the ^13^C analyses were incorporated into a COnstraints-Based Reconstruction and Analysis (COBRA) genome-scale metabolic model. COBRA (10, 11) is flux balance analysis (FBA) based on stoichiometry equations describing a genome-scale model network that predicts flux distributions to achieve optimization of an objective function. It is an approach complementary to ^13^C based flux analysis since the network covered in FBA is much broader. However, the assumption that the network is operating to provide optimization with respect to a specific objective goal may not be correct, and multiple possible metabolic configurations could lead to the same value of objective function. Such issues can further lead to a predicted flux distribution differing from the experimentally validated ^13^C metabolic flux analysis (MFA) distribution, leading to less accurate predictions. The ^13^C analysis data provided additional constraints to better predict flux distribution of central metabolism for growth on methanol, while quantification of secreted products was used to further improve the performance of the COBRA model.

## RESULTS

### Targeted metabolomics analysis shows systematic flux distribution shift for growth on methanol compared to methane.

**FIG 2:**
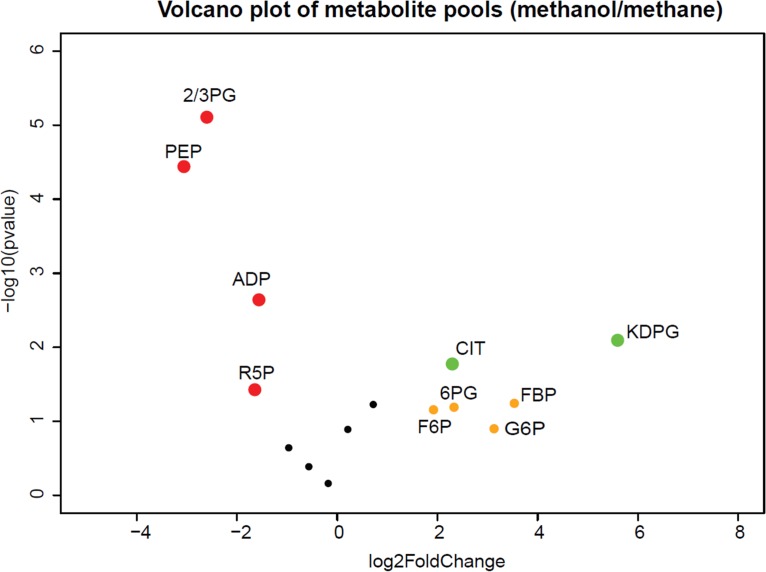
Volcano plot of metabolite pools for methanol-grown versus methane-grown cultures with fold changes and *P* values. The changes of metabolite pools indicated altered flux through those metabolite nodes. Red dots represent metabolites with decreased pool size and statistically significant *P* values. Green dots represent metabolites with increased pool size and statistically significant *P* values. Orange dots represent metabolites with increased pool size and broad *P* value ranges.

Metabolomics gives a direct view of cell physiology as a result of the integrated effect of gene expression and regulation in the highly interconnected metabolic network (12). Changes in the intracellular level of metabolites indicate metabolite nodes with altered flux. Targeted metabolomics analysis was performed for both methane cultures and methanol cultures in vials during the exponential-growth phase to locate metabolite nodes that respond to the substrate shifts. Each condition corresponded to at least three biological replicates. Intermediate metabolites were extracted and quantitated under both conditions. The levels of metabolites were normalized to both an internal standard (^13^C_4_ succinate) and cell biomass. As seen in Fig. 2, the volcano plot shows the difference in pool size for methanol-grown versus methane-grown cultures with log2-fold change and log10 (*P* values). The following metabolites showed significant pool size increases in methanol culture: glucose 6-phosphate (G6P), F6P, FBP, citrate, 6PG, and KDPG, with KDPG showing the largest increase of approximately 60-fold. The results seen with the first four metabolites indicate flux through the glycogen synthesis pathway, the upper EMP pathway, and the tricarboxylic acid (TCA) cycle, while the results seen with the latter two metabolites indicate flux through the ED pathway. Three metabolites showed pool size decreases: phosphoenolpyruvate (PEP), 2-phosphoglycerate (2PG) plus 3-phosphoglycerate (3PG) (measured as a total pool), and R5P, intermediates of the lower EMP pathway and the RuMP cycle. The sizes of the pools of the TCA cycle intermediates malate, succinate, and 2-oxo-glutarate did not change significantly.

These results show that most of the metabolites in core carbon metabolism had changed pool sizes under conditions of growth on methanol compared to methane, which indicates a systematic shift of flux distribution during growth on methanol (13).

### Expression of central metabolism genes showed minor differences.

Transcriptome sequencing (RNA-seq) data were used to identify expression differences of genes involved in central metabolism for cultures grown on methane compared to methanol. RNA samples were obtained from cells taken from fed-batch bioreactor experiments performed with either methane or methanol as the sole carbon source (4). Table 1 includes the genes in central metabolism with fold change values and *P* values (cutoff value = 0.05) comparing methane and methanol cultures. The gene expression log2-fold changes in expression of these genes were in the range of 0.3 to 2.8. However, most of these genes showed modest changes (upregulated or downregulated less than 2-fold), suggesting that the change in metabolism was not due to large changes in transcription profiles. It is noteworthy that despite large differences in glycogen content, transcription of genes involved in synthesis of glycogen (*glgA1* and *glgA2*) actually decreased slightly in cultures grown on methanol versus methane, and although the glycogen phosphorylase genes involved in glycogen degradation had a *P* value too high to report (0.08), this transcription also did not appear to be altered (see Table S1 in the supplemental material).

**TABLE 1:** Fold change of gene expression levels in core metabolism comparing growth on methanol to growth on methane with *P* values of less than 0.05

Locus tag	Gene	Product	Fold change	Log2 fold change	*P* value
MBURv2_210062		Lactate/malate dehydrogenase	0.36	−1.48	0.00
MBURv2_130865	*fumC*	Fumarate hydratase (fumarase C), aerobic class II	0.42	−1.25	0.01
MBURv2_20327	*pykA*	Pyruvate kinase	0.45	−1.17	0.04
MBURv2_210058	*ald*	Alanine dehydrogenase	0.47	−1.08	0.00
MBURv2_130928		NADH:ubiquinone dehydrogenase subunit, associated with formate dehydrogenase	0.57	−0.921	0.01
MBURv2_130931	*fdhD*	Formate dehydrogenase associated protein	0.58	−0.79	0.01
MBURv2_130929		NADH: ubiquinone dehydrogenase subunit, associated with formate dehydrogenase	0.66	−0.66	0.01
MBURv2_130613	*sps*	Sucrose-phosphate synthase	0.60	−0.75	0.00
MBURv2_130610	*ams*	Amylosucrase	0.63	−0.66	0.01
MBURv2_210335		6-Phosphogluconate dehydrogenase NAD-binding	0.67	−0.59	0.00
MBURv2_210177	*glgA1*	Glycogen synthase	0.68	−0.55	0.00
MBURv2_210185	*glgA2*	Glycogen synthase (*P* value too low to include; data in Table S1)			
MBURv2_130310	*mdh*	Malate dehydrogenase	0.69	−0.54	0.01
MBURv2_160480	*fda*	Fructose-1,6-bisphosphate aldolase, class II	0.70	−0.52	0.03
MBURv2_120045	*mtnB*	Methylthioribulose-1-phosphate dehydratase	0.71	−0.50	0.02
MBURv2_130929		Formate dehydrogenase alpha subunit (Mo-enzyme)	0.74	−0.44	0.01
MBURv2_80101	*sdhA*	Succinate dehydrogenase, flavoprotein subunit	0.76	−0.40	0.01
MBURv2_20302	*pps*	Phosphoenolpyruvate synthase	0.79	−0.34	0.02
MBURv2_190108		Putative glyceraldehyde-3-phosphate dehydrogenase	0.81	−0.31	0.00
MBURv2_80100	*sdhB*	Succinate dehydrogenase, FeS subunit	0.83	−0.26	0.01
MBURv2_160358	*icd*	Isocitrate dehydrogenase (NADP)	0.86	−0.21	0.04
MBURv2_80063	*tkt*	Transketolase domain protein	1.09	0.12	0.02
MBURv2_160221	*zwf*	Glucose-6-phosphate dehydrogenase	1.11	0.16	0.03
MBURv2_160313	*rmpB*	3-Hexulose-6-phosphate isomerase	1.23	0.29	0.00
MBURv2_160305	*rmpB*	3-hexulose-6-phosphate isomerase	1.24	0.31	0.00
MBURv2_160244	*rpe*	d-Ribulose-5-phosphate 3-epimerase	1.25	0.32	0.03
MBURv2_210071	*oadB*	Putative oxaloacetate decarboxylase beta chain	1.37	0.45	0.04
MBURv2_60009	*tpiA*	Triosephosphate isomerase	1.38	0.46	0.01
MBURv2_210199	*fchA*	Methenyltetrahydrofolate cyclohydrolase	1.40	0.48	0.03
MBURv2_130012	*pdhB*	Pyruvate dehydrogenase E2 component; dihydrolipoamide acetyltransferase	1.41	0.49	0.04
MBURv2_130389	*sucC*	Succinyl-CoA synthetase, beta subunit	1.46	0.55	0.01
MBURv2_130008	*edd*	6-Phosphogluconate dehydratase	1.49	0.58	0.03
MBURv2_130011	*aceE*	Pyruvate dehydrogenase, decarboxylase component E1, thiamin-binding	1.51	0.60	0.01
MBURv2_130313	*glyA*	Serine hydroxymethyltransferase	1.59	0.67	0.00
MBURv2_130401	*pfp*	Pyrophosphate–fructose 6-phosphate 1–phosphotransferase	1.68	0.75	0.00
MBURv2_210131	*pgk*	Phosphoglycerate kinase	1.68	0.75	0.01
MBURv2_20405	*eno*	Enolase	1.87	0.90	0.01
MBURv2_160304	*rmpA*	3-Hexulose-6-phosphate synthase	1.91	0.94	0.00
MBURv2_130302	*sgaA*	Serine-glyoxylate aminotransferase	1.93	0.95	0.01
MBURv2_160312	*rmpA*	3-Hexulose-6-phosphate synthase	1.94	0.95	0.00
MBURv2_160308	*rmpA*	3-Hexulose-6-phosphate synthase	1.94	0.96	0.00
MBURv2_130299	*sucC*	Succinyl-CoA synthetase, beta subunit	2.05	1.03	0.01
MBURv2_30146	*leuB*	3-Isopropylmalate dehydrogenase	2.30	1.20	0.00
MBURv2_50413	*gpmI*	2,3-Bisphosphoglycerate-independent phosphoglycerate mutase	2.82	1.50	0.00

10.1128/mBio.00406-19.3 TABLE S1 RNA-seq results. Download Table S1, XLS file, 0.8 MB. Copyright © 2019 Fu et al. 2019 Fu et al. This content is distributed under the terms of the Creative Commons Attribution 4.0 International license.

### Supernatant metabolite profiles confirmed high formate excretion levels.

Although excretion products have been reported for fed-batch bioreactor cultures of M. buryatense 5GB1 grown on methanol (4), no data are available for vial growth cultures. Vial cultures grown on either methane or methanol to mid-log phase were processed as described in Materials and Methods and analyzed using nuclear magnetic resonance (NMR) to obtain a profile for extracellular metabolites. As shown in Table 2, for methanol-grown cultures, 8.85 ± 0.29 mmol formate/gram cell dried weight (gcdw) was excreted into the supernatant together with much smaller amounts of acetate and lactate. This formate level is about 5-fold higher than that seen with methane-grown cultures, a trend in keeping with previously reported bioreactor results (4). This extracellular metabolite profile was then used for constraining the genome-scale model for better flux prediction. The ethanol concentration in the supernatant was also monitored (see Materials and Methods). The specific product yields determined on the basis of the amount of substrate utilized were then calculated, showing that about 10% of the methanol used was excreted as formate.

**TABLE 2:** Extracellular product yield and biomass for cultures grown on methanol or methane^*a*^

Yield	Methanol-grown cultures	Methane-grown cultures
Product (mmol/gcdw)		
Formate	8.85 ± 0.29	1.92 ± 0.51
Acetate	0.18 ± 0.13	0.13 ± 0.01
Lactate	0.01 ± 0.00	0.01 ± 0.00

Substrate (mmol/mmol methanol consumed)		
Formate	0.09 ± 0.01	NA
Acetate	1.00 × 10^−3^ ± 3.66 × 10^−4^	NA
Lactate	5.92 × 10^−5^ ± 2.92 × 10^−5^	NA

a Values represent at least two replicates for methanol-grown cultures and methane-grown cultures. NA, not applicable.

### ^13^C tracer analysis-elucidated relative flux ratio downstream of the PEP node.

As described previously for methane-grown cultures (14), steady-state ^13^C analysis cannot be used to resolve a significant portion of core metabolism, as the intermediates of core metabolism upstream of and including PEP become fully labeled. However, it can be used to resolve relative flux contributions downstream of the PEP and pyruvate nodes due to CO_2_ incorporation. In this study, the same procedure was carried out using ^13^C methanol as the tracer substrate. As expected from the results from the methane-grown cultures, the levels of flux occurring through the RuMP cycle, the EMP pathway, and the ED pathway could not be distinguished (14). The mass isotopomer distributions (MIDs) of 2 key metabolites are shown in Fig. 1b (a full list is shown in Table S2). These results clearly show that malate and threonine (an indicator of the presence of oxaloacetic acid [OAA], which is poorly measured under these conditions [14]) have similar labeling patterns, with M + 3 levels being higher than M+4 levels, suggesting that the major flux with respect to *de novo* production of OAA and malate was occurring through carboxylation reactions from pyruvate and PEP (Fig. 1a). These results also suggest little to no flux contribution from the oxidative TCA cycle to *de novo* malate production. TCA cycle contribution was investigated by applying ^13^C analysis to both Δ*fumC* and Δ*fumA* mutants. As reported previously for methane-grown cultures (14), a Δ*fumA* mutant disrupted the oxidative TCA cycle. The consistent labeling pattern of OAA and malate for the Δ*fumA* mutant versus the wild-type (WT) strain supported the hypothesis of minor flux contribution to malate from the TCA cycle.

10.1128/mBio.00406-19.4 TABLE S2 MIDs of key metabolites. Download Table S2, XLSX file, 0.02 MB. Copyright © 2019 Fu et al. 2019 Fu et al. This content is distributed under the terms of the Creative Commons Attribution 4.0 International license.

**FIG 3:**
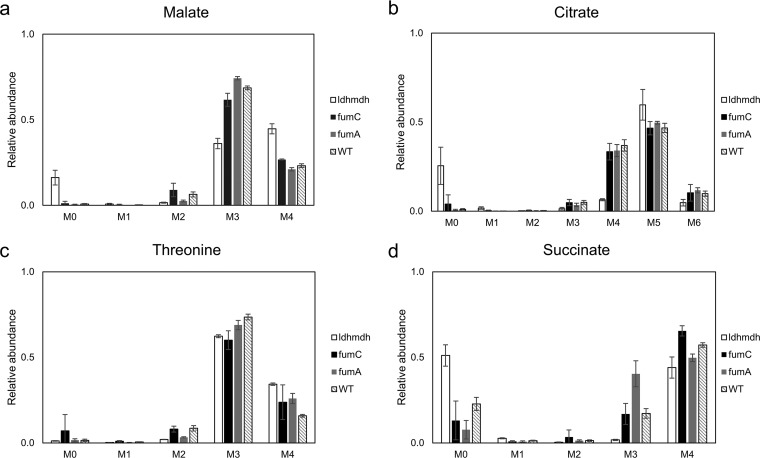
MID of central metabolites of the WT strain and *ΔfumA*, *ΔfumC*, and *Δldh Δmdh* mutants. (a) Malate, where the *Δldh Δmdh* mutant had higher M+4 values than the rest of the strains. (b) Citrate, where the *Δldh Δmdh* mutant had decreased M+4 values. (c) Threonine, whose precursor is OAA, showed consistent labeling patterns among all strains. (d) Succinate, where the *Δldh Δmdh* mutant showed a labeling pattern distinct from those seen with the other strains.

To observe the direct effect of the carboxylation reactions in OAA and malate synthesis, the interconversion of OAA and malate was blocked by mutation. In M. buryatense 5GB1, two genes are predicted to be involved in malate dehydrogenase activity, *ldh* (*MBURv2_210062*) and *mdh* (*MBURv2_130310*). A double mutant of these genes was generated, and ^13^C analysis was performed. The selective MIDs of malate, citrate, threonine and succinate were determined (Fig. 3), and the full list can be found in Table S2. The labeling pattern of threonine for WT and Δ*ldh*Δ*mdh* remains the same, confirming the dominance of carboxylation reactions in producing OAA. Malate showed a different labeling pattern in the Δ*ldh*Δ*mdh* strain, with a higher proportion of M+4 than M+3. In this strain, the conversion of OAA to malate was disrupted, reducing the source of M+3 label, and making the small contribution of the oxidative TCA cycle to the M+4 of malate a relatively greater proportion of the pool. Taken together, these results support the conclusion that OAA and malate are mainly generated from carboxylation of C3 compounds, not from the TCA cycle during growth on methanol.

The CO_2_ pool was also determined as previously described (14) and found to be 76% unlabeled (diffusion from the extracellular source) and 24% labeled (from ^13^C methanol oxidation to ^13^CO_2_).

**FIG 4:**
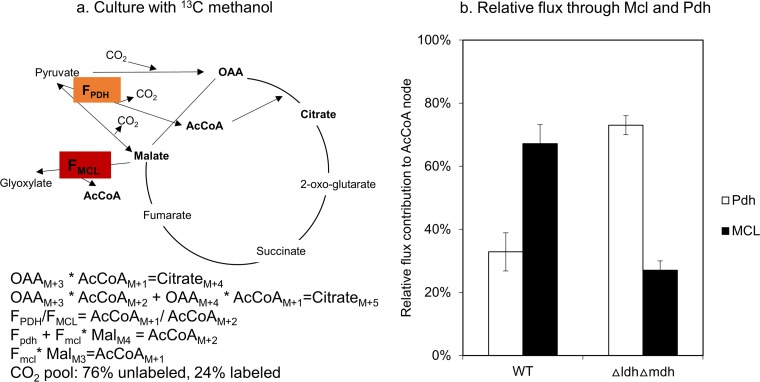
Quantitation method and result of relative flux ratio between F_PDH_ and F_MCL_. (a) Equations describing the labeling patterns of intermediate metabolites as well as relative fluxes from F_PDH_ and F_MCL_ to AcCoA. (b) Relative fluxes through PDH and MCL in both the WT strain and the *Δldh Δmdh* mutant.

Citrate would be expected to have labeling mainly in M+5 and M+6, if acetyl coenzyme A (AcCoA) were fully labeled in the acetyl moiety. However, it was found to have significant M+4, suggesting that AcCoA has two sources, from pyruvate (fully labeled, generating M+5 and M+6) through pyruvate dehydrogenase and from malate through malyl-CoA lyase (partially labeled, generating M+4) (Fig. 1a). The relative flux contribution to *de novo* production of AcCoA was then determined using the method described in Fig. 4a. As shown in Fig. 4b, the relative flux contribution to AcCoA in WT is calculated to be 33% from pyruvate, presumably from pyruvate dehydrogenase (PDH) and 67% from malyl-CoA lyase (MCL). For the Δ*ldh*Δ*mdh* double-knockout mutant strain, the relative contribution to AcCoA was also calculated as shown in Fig. 4b. The contribution from PDH increased to 73% while the contribution from MCL decreased to 27%. This relative flux contribution between these two pathways was also incorporated into the genome-scale model as additional constraints.

### Modeling growth on methanol suggests a possible configuration consistent with experimental results.

A genome-scale model for growth at low O_2_ previously published (5) was modified to reflect methanol metabolism by adding a methanol transport reaction (see supplemental material Table S5). Other modifications were made as follows. In the original model, the flux ratio of EMP and ED is constrained by 3:1. This constraint is removed for methanol metabolism since the relative ratio has not been determined for this growth condition. We assumed that the biomass composition other than glycogen was similar to that for cultures grown on methane, which leads to an unchanged stoichiometry of precursors included in the biomass equation. Glycogen content in methanol-grown cells has been previously measured as 42% of the biomass dry weight (4).

10.1128/mBio.00406-19.7 TABLE S5 List of metabolic model reactions, metabolites, and predicted flux distributions. Download Table S5, XLSX file, 0.1 MB. Copyright © 2019 Fu et al. 2019 Fu et al. This content is distributed under the terms of the Creative Commons Attribution 4.0 International license.

Specific methanol uptake was measured in vial cultures and found to be 19.3 (±1.01) mmol methanol/(gcdw·h). All of the flux balance analyses done in this study were optimized to maximize biomass growth rate. With the control model, substrate uptake rate, measured extracellular product rates, glycogen content and extracellular polymeric substance (EPS) content (assumed to be similar to methane-grown cultures [4]) were constrained. Further, we used the same values of ATP maintenance energy as those previously reported (5): the growth-associated and non-growth-associated ATP maintenance energy levels were 54.35 mmol/gcdw and 8.39 mmol/(gcdw·h), respectively. We also tested a series of values for ATP maintenance requirements, and the results confirmed that the numbers reported above gave reasonable growth rates at ∼0.2 h^−1^ (see Fig. S1 in the supplemental material).

10.1128/mBio.00406-19.2 FIG S1 Assessment of non-growth-associated and growth-associated ATP maintenance values. (a) A three-dimensional plot of predicted growth rates in response to different non-growth-associated ATP maintenance (NGA ATPM) and growth-associated ATP maintenance (GA ATPM) fluxes. (b) Contour plot of growth rate, NGA ATPM, and GA ATPM. The numbers on the contour lines represent growth rates. Download FIG S1, TIF file, 1.2 MB. Copyright © 2019 Fu et al. 2019 Fu et al. This content is distributed under the terms of the Creative Commons Attribution 4.0 International license.

**FIG 5:**
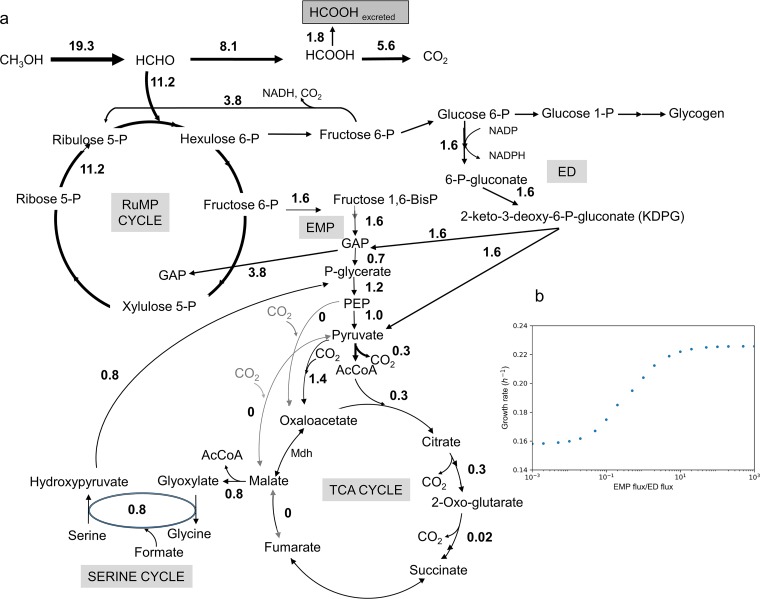
(a) Flux distribution predicted in COBRApy with the same constraints as those applied in the ED/EMP_constrained model (Table 3; see also Table S5). (b) Sensitivity analysis of EMP flux/ED flux ratio to growth rate. The results suggest that the ratio impacts the growth rate only when the value is between 0.1 and 10.

Several cases were simulated in the model with COBRApy, and the results are summarized in Table 3. The optimal growth rate under the “control” condition is predicted to be 0.248 h^−1^, which is higher than the range of measured growth rates (0.17 to 0.2 h^−1^) in vials or fed-batch bioreactor (4). The model was then further constrained based on the flux ratio determined through ^13^C tracer analysis (Fig. 3 and 4). The predicted growth rate dropped to 0.241 h^−1^ with an incomplete TCA cycle (fumarase reaction was set to 0). Finally, with the ratio of MCLA1/PDH = 3 constraint, the predicted growth rate dropped to 0.226 h^−1^. This represented a total 9% decrease in biomass flux with additional constraints from ^13^C tracer analysis. The impact of the flux ratio between EMP and ED on predicted growth rate was also evaluated based on the model with ^13^C results integrated. As shown in Fig. 5b, decreasing EMP/ED flux ratios reduced the value of the predicted growth rate. An EMP/ED flux ratio of 1:1 yielded a good fit to the experimental results (Table 3). The flux map generated from the last scenario is shown in Fig. 5a.

**TABLE 3:** Result summary for FBA

Models with different constraints	Growth rate (h^−1^)	O_2_/methanol consumption ratio	Biomass yield (g biomass/ g methanol)	Notes
Control	0.248	0.500	0.398	EPS and glycogen account for 10% and 42% of the total biomass, respectively; methanol uptake rate = 19.3 mmol/(gcdw·h); formate production rate = 1.82 mmol/(gcdw·h); O_2_/methanol consumption ratio ≥ 0.5
TCA_constrained	0.241	0.500	0.388	Based on the control model; set alpha-ketoglutarate dehydrogenase flux ≥ succinyl-CoA synthetase flux and fumarase flux = 0 mmol/(gcdw·h)
MCL1A/PDH_constrained	0.226	0.561	0.363	Based on the TCA_constrained model; set MCL1A flux/PDH flux = 3:1
ED/EMP_constrained	0.204	0.613	0.328	Based on the MCL1A/PDH_constrained model; set ED flux/EMP flux = 1:1
Exptl results	0.205 ± 0.014	NA	0.332 ± 0.006	The experimental results were based on two biological replicates; the measured methanol uptake rate is 19.3 ± 1.01 mmol/(gcdw·h), and the measured formate production rate is 1.82 ± 0.19 mmol/(gcdw·h)

**FIG 6:**
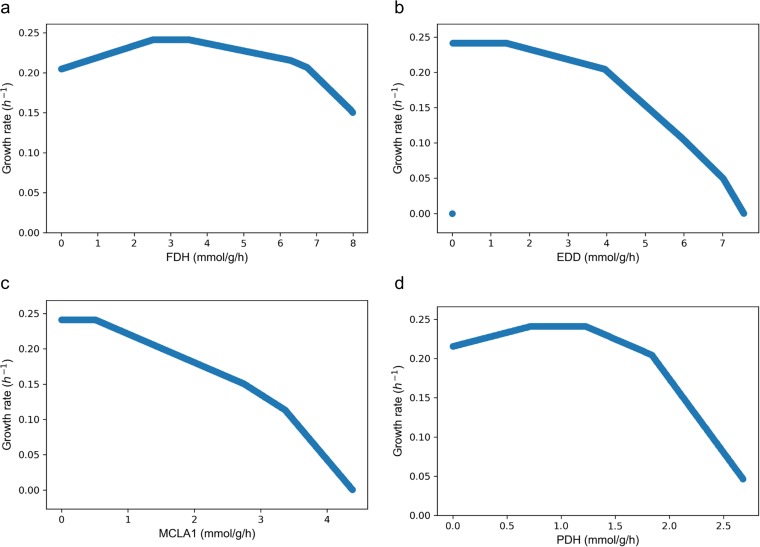
Robustness analysis of FDH (a), EDD (b), MCLA1 (c), and PDH (d) fluxes versus the growth rate. The constraints were the same as those described for the control model shown in Table 3.

Furthermore, we evaluated how fluxes through branch points, in particular, the FDH, 6-phosphogluconate dehydratase (EDD, an enzyme in the ED pathway), PDH, and MCLA1 pathways, impacted the predicted growth rate using robustness analysis. An optimum range of about 2.5 to 3.5 mmol/(gcdw·h) is predicted for FDH flux (Fig. 6a). Below or beyond that range, the growth rate is predicted to be suboptimal. This prediction is in line with our previous experimental finding that a FDH-negative mutant has a severe growth defect and excretes more formate (15). As shown in Fig. 6b, flux through the ED pathway does not impact the predicted growth rate until the value is higher than 1.5 mmol/(gcdw·h). Beyond that point, the growth rate correlates negatively with higher flux through the ED pathway. The flux through the MCL pathway (MCLA1 reaction) shows a trend similar to that shown by the ED pathway (Fig. 6c), as a flux larger than 0.5 mmol/(gcdw·h) reduced the predicted growth rate. Finally, the PDH flux versus the biomass growth rate has a pattern similar to that seen with FDH, with the optimal range being from 0.7 to 1.3 mmol/(gcdw·h) (Fig. 6c). All four pathways have maximum acceptable values beyond which no feasible solutions could be found. For instance, the FDH flux has the broadest range, from 0 to 8 mmol/(gcdw·h), while the PDH flux can be no more than 3 mmol/(gcdw·h).

## DISCUSSION

**FIG 7:**
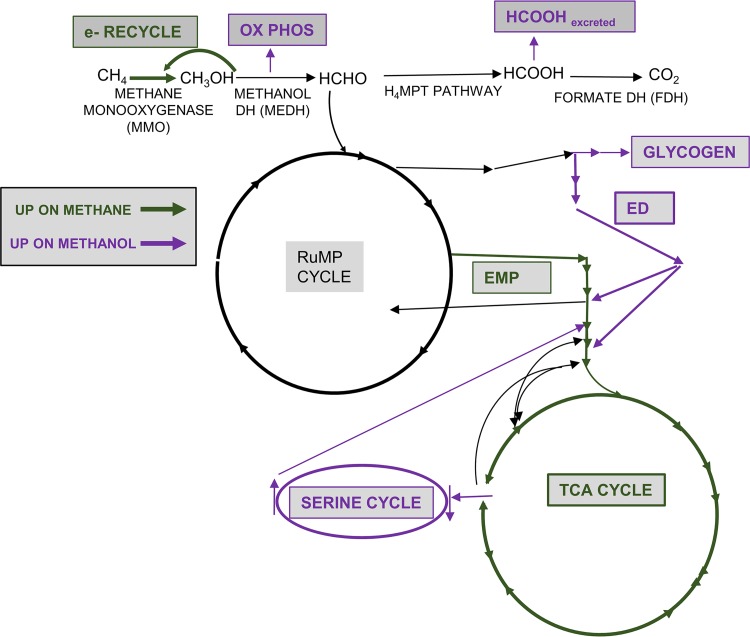
Summary of differences between methane and methanol metabolism. Green arrows show metabolic processes whose levels were increased in cultures grown on methane compared to methanol, and purple arrows show those whose levels were increased in cultures grown on methanol compared to methane.

Previous studies (4, 5) raised the possibility that metabolism in M. buryatense 5GB1 during growth on methanol could be significantly different from that on methane, especially with regard to ATP and NADH utilization. In this study, we used a variety of approaches to assess metabolism during growth on these two substrates, and we identified major differences (Fig. 7).

First, the pools of several metabolites in core metabolism showed significant alterations that were consistent with major shifts in fluxes. The most striking of these was KDPG, an intermediate of the ED pathway, which showed a large increase. Coupled to significant decreases in EMP pathway intermediates PEP and 2PG plus 3PG, these results suggest a major change in the ratio of flux through the EMP and ED pathways. This concept was supported by modeling, which showed that increasing fluxes through the ED pathway helped align model predictions with measured growth rates. Flux through the ED pathway generates less ATP than flux through the EMP pathway, which is in keeping with the prediction that the ATP generated as a result of methanol dehydrogenase activity would result in a lower requirement for ATP production in other parts of metabolism. Other changes are predicted to affect NADH balance. As reported previously (4), levels of excreted formate were much higher in methanol-grown cultures than in methane-grown cultures, amounting to about 10% of the total methanol utilized. Such a result is consistent with lower NADH consumption needs, since the formate that is excreted is not oxidized to CO_2_ and does not generate NADH by this reaction (Fig. 1).

The flux distributions among the C3-C4 interconversion reactions, especially the relative flux distributions occurring through pyruvate dehydrogenase and malyl-CoA lyase, were significantly different for growth on methanol compared to methane. Malyl-CoA lyase and the serine cycle have been proven to be inactive in cultures grown on methane; thus, pyruvate dehydrogenase is the major pathway contributing to AcCoA synthesis. In contrast, results from our ^13^C labeling experiments confirmed that malyl-CoA lyase plays a more important role in AcCoA production under methanol conditions, contributing much more than the PDH reaction. Robustness analysis also shows that PDH and MCL1A fluxes show different correlations with the growth rate. Together, these results suggest that these two pathways could be control points of the network for responding to methanol versus methane as a substrate.

Our results show that the oxidative TCA cycle contributes little to *de novo* malate synthesis, in contrast to growth on methane, under which conditions a full oxidative cycle operates to produce malate and NADH (14). This suggests that the main function of the TCA cycle during growth on methanol is to provide precursors for *de novo* biosynthesis, decreasing the amount of NADH generated by this pathway.

Our results show a major set of changes in flux through the central metabolic pathways when methanol was the growth substrate, but the RNA-seq results show relatively small changes in transcription for the key genes involved in these reactions, highlighting the importance of directly measuring metabolites. This result is especially surprising for glycogen synthesis and degradation genes, given the large change in glycogen content, and for the genes specific to the ED pathway, given a similar large change in flux. This suggests that the changes in metabolite pools and fluxes likely represent results of posttranscriptional regulation at the protein effector level or the enzyme effector level or both. A similar result has been found in the serine cycle methanol utilizer Methylobacterium extorquens AM1 during the transition from growth on succinate to growth on methanol (16) and in the methanotroph Methylomicrobium alcaliphilum 20Z^R^ in comparing growth rates with and without lanthanides (17).

As shown in this study, integrating ^13^C results into the genome-scale model provides additional constraints to the model, which redefines the solution space for predictions giving a better fit to the experimental measurements. A recent effort has been made to develop a method to constrain genome-scale models with ^13^C labeling data (18) and to eliminate the need to assume an objective function for optimization. However, we foresee the limitation of this method in C1 networks based on the same rationale for the limitation of the well-established ^13^C MFA method, which is the indistinguishable labeling patterns of many key intermediates in the RuMP cycle.

This analysis of M. buryatense 5GB1 growth on methanol extends our understanding of core metabolism in this methanotroph, demonstrating the major differences in metabolism of the two one-carbon substrates methane and methanol. The insights presented here and captured in the improved model can now serve as an improved platform for future strain engineering for growth on either methane or methanol, taking into account the differences for each substrate.

## MATERIALS AND METHODS

### Cell culture for ^13^C labeling experiment and for phenotypic characterization.

For growth curve experiments, liquid precultures were grown in 25-ml tubes in modified nitrate mineral salts medium (NMS2) (6) with 0.2% methanol at 30°C and 200 rpm; the tubes were sealed with rubber stoppers and aluminum seals (Wheaton, Millville, NJ, USA). Precultures were then used to inoculate new 25-ml tubes with a starting optical density at 600 nm (OD_600_) of 0.01 for growth rate experiments as well as ^13^C analysis. NMS2 was used for liquid culture as described previously (19).

For ^13^C labeling experiments, precultures were grown in 25-ml tubes with 0.2% methanol for 18 h. The tube cultures were then used to inoculate 50 ml fresh medium into 250-ml serum bottles (with a starting OD_600_ of 0.01). Cells were harvested at an OD_600_ of approximately 0.3 to 0.6, when the culture was at both the isotopic steady state and the metabolic steady state (20). All strains used in this study are listed in the supplemental materials (see Table S3 in the supplemental material).

10.1128/mBio.00406-19.5 TABLE S3 Strain list. Download Table S3, XLSX file, 0.01 MB. Copyright © 2019 Fu et al. 2019 Fu et al. This content is distributed under the terms of the Creative Commons Attribution 4.0 International license.

### Measurements of methanol and formate concentrations in culture supernatants.

Methanol concentrations were measured by using a commercial methanol assay kit (Biovision, Inc., CA, USA). Procedures were followed by the instructions provided in the kit. Formate concentrations were measured by the use of a Dionex ICS-5000 Ion Chromatography system (Thermo Fisher Scientific, Waltham, MA) equipped with a Dionex IonPac ICE-AS6 column (Thermo Fisher Scientific, Waltham, MA) (9 by 250 mm). The eluent, 0.5 mM heptafluorobutyric acid, was used at a flow rate of 1.0 ml/min and a temperature of 30°C, and the regenerant, 5 mM tetrabutylammonium hydroxide, was used at a flow rate of 3.5 ml/min and the same temperature. All the reagents were purchased from Sigma-Aldrich (St. Louis, MO, USA).

### RNA-seq analysis.

Samples for RNA-seq analysis were taken from bioreactor experiments performed with batch cultures grown on either methane or methanol under pH control and continuous methane and airflow conditions, which have been described previously (4). Samples were taken at the late log phase. RNA was isolated and RNA-seq data analysis was carried out as described previously (5). Normalized counts and computed pairwise fold changes for the RNA-seq experiments are available in Table S1. These RNA-seq data have also been submitted to the Gene Expression Omnibus (GEO) database under accession number GSE110541.

### ^13^C labeling pattern of metabolite measurements and targeted metabolomics analysis using LC/MS-MS.

The cell quenching procedure was carried out with fast filtration and hot water extraction as previously described (21). Briefly, cell cultures were quenched using fast filtration and were saved in 50-ml Falcon tubes submerged in liquid nitrogen. The collected samples were lyophilized for 12 h to remove extra medium. The hot water extraction protocol was used to extract intracellular metabolites as described previously (21). Briefly, 20 ml of boiling water was added to 50-ml Falcon tubes, andthen the tube was placed into a hot water bath at 100°C for 20 min. The tubes were placed then on ice for 30 min. The cell extracts were centrifuged at 4°C and 3,000 × *g* for 30 min to remove cell biomass. The supernatant was transferred into new tubes and was fast frozen using liquid nitrogen. The cell lysates were lyophilized and concentrated into 50 µl water. The reconstituted samples were centrifuged with a filter (Spin-X centrifuge tube filters; Corning Inc, NY) (pore size, 0.22 μm) and then kept at −20°C until liquid chromatography/mass spectrometry (LC/MS) analysis for both ^13^C labeling pattern measurements and the targeted metabolomics study. Waters Xevo mass spectrometry (Waters Corporation, Milford, MA) was used with an ultraperformance liquid chromatography (UPLC) system for detection of labeling patterns of metabolites. Multiple-reaction monitors (MRM) were set up for each metabolite of interest. This information is also included in the supplemental material (see Table S4). Similarly, for targeted metabolomics, an internal standard of 25 μM succinate-^13^C4 and l-alanine-^13^C3 ^15^N (Sigma-Aldrich, St. Louis, MO) was spiked to the cell extract for relative quantification of metabolite pools. Multiple reaction monitors were set up for a full set of metabolites, as listed in the supplemental materials.

10.1128/mBio.00406-19.6 TABLE S4 List of MRM reactions. Download Table S4, XLSX file, 0.01 MB. Copyright © 2019 Fu et al. 2019 Fu et al. This content is distributed under the terms of the Creative Commons Attribution 4.0 International license.

Extracellular metabolite secretion was measured using ^1^H NMR with a previously described protocol (4).

### Calculation of relative flux distribution in acetyl-CoA node.

Acetyl-CoA (AcCoA) has differentiated labeling patterns depending on the pathway for synthesis. Based on current understanding of central carbon metabolism in M. buryatense 5GB1, AcCoA could be synthesized in two different ways. First, it could be produced from pyruvate through pyruvate dehydrogenase; second; it could be produced from malyl-CoA through malyl-CoA lyase. Route 1 leads to fully labeled AcCoA. Route 2 produces carbon-labeled AcCoA first. MIDs of AcCoA are calculated from citrate as well as threonine (serving for OAA) since AcCoA cannot be measured directly due to the small pool amount. The following equations were then developed to estimate the relative flux amounts in the AcCoA node between route 1 and route 2.

$${\text{OAA}}_{M+3}*{\text{AcCoA}}_{M+1}={\text{citrate}}_{M+4}$$

$${\text{OAA}}_{M+3}*{\text{AcCoA}}_{M+2}+{\text{OAA}}_{M+4}*AcCoA_{M+1}={\text{citrate}}_{M+5}$$

$$F_{\text{PDH}}/F_{\text{MCL}}={\text{AcCoA}}_{M+1}/{\text{AcCoA}}_{M+2}$$

$$F_{\text{PDH}}+F_{\text{MCL}}*{\text{Mal}}_{M4}={\text{AcCoA}}_{M+2}$$

$$F_{\text{MCL}}*{\text{Mal}}_{M3}={\text{AcCoA}}_{M+1}$$

### Flux balance analysis and robustness analysis.

Genome-scale model simulation was done in COBRApy (22) with an updated model. The updated model is shown in the supplemental materials (Table S5). Robustness analysis and addition of the flux ratio constraints to the model were done with in-house-developed python scripts; both scripts are shown in the supplemental materials. In the robustness analysis, we tested how the flux of a certain reaction correlated with the growth rate by gradually increasing the value corresponding to the flux through this reaction from 0 to a value that generated no feasible solution.

### Data availability.

RNA-seq data have been deposited in the Gene Expression Omnibus (GEO) database under accession number GSE110541. The python scripts used in this study are available in the supplemental material (see Text S1 in the supplemental material).

10.1128/mBio.00406-19.1 TEXT S1 Python scripts used in this study. Download Text S1, DOCX file, 0.1 MB. Copyright © 2019 Fu et al. 2019 Fu et al. This content is distributed under the terms of the Creative Commons Attribution 4.0 International license.
