# Supplementary figures and images for: Core Metabolism Shifts during Growth on Methanol versus Methane in the Methanotroph Methylomicrobium buryatense 5GB1
Source: mBio. 2019 Apr 9;10(2):e00406-19. doi: 10.1128/mBio.00406-19 (PMC6456754; doi:10.1128/mBio.00406-19)

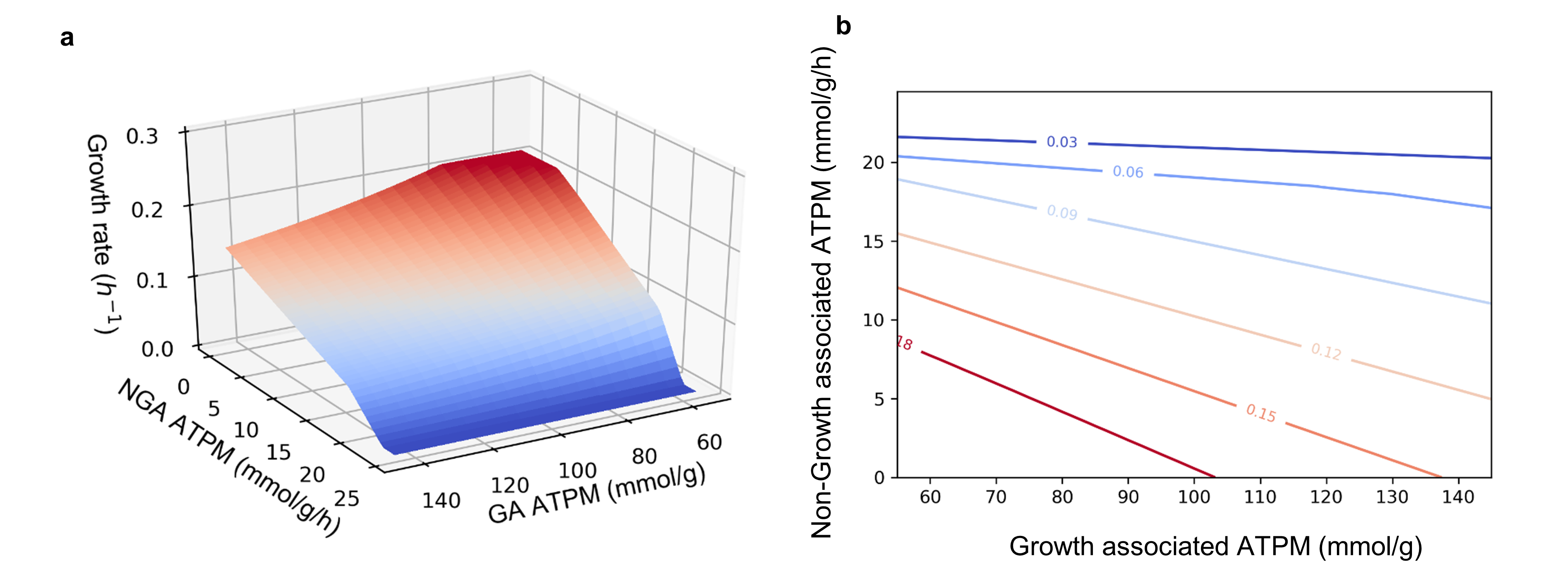

Supplement: FIG S1 [file mBio.00406-19-sf001.tif]
