# Supplementary material for: Core Metabolism Shifts during Growth on Methanol versus Methane in the Methanotroph Methylomicrobium buryatense 5GB1
Source: mBio. 2019 Apr 9;10(2):e00406-19. doi: 10.1128/mBio.00406-19 (PMC6456754; doi:10.1128/mBio.00406-19)
Supplement: TEXT S1 [file mBio.00406-19-s0001.docx]

Python Scripts

1. Robustness analysis

#function robustnessanalysis

'''

Inputs:

    model: already imported into cobra

    controlRXN: string of a reaction name

    npoints: positive integer

    objective function: string of a reaction name

    objType either maximize or minimize

Output:

    matplotlib figure

'''

import numpy as np

import cobra

from cobra import Reaction

import matplotlib.pyplot as plt

from copy import deepcopy

def robustnessanalysis(model, controlRXn, npoints, obj_function, objType):

    # step 1, check if the control RXn is in the model

    if model.reactions.has_id(controlRXn):

        #Initialize the model by deleting all exiting objetive function

        model_init=model.copy()

        Init_obj = model_init.objective

        Init_obj.clear()

        model_init.objective = Init_obj

        tempmodel=model_init.copy()

        #after initiliation, set the objective function based on the input objective function

        model_init.objective=obj_function

        # create a temp model to get the low bound and up bound of control_rxn

        tempmodel.reactions.get_by_id(controlRXn).objective_coefficient = 1

        obj_min = "minimize"

        obj_max = "maximize"

        sol_min = tempmodel.optimize(objective_sense=obj_min)

        sol_max = tempmodel.optimize(objective_sense=obj_max)

        controlFlux = np.linspace(sol_min.f, sol_max.f, npoints)

        objFlux = list()

        n = len(controlFlux)

        control_rx = model_init.reactions.get_by_id(controlRXn)

        for i in range(0, n):

            control_rx.lower_bound = controlFlux[i]

            control_rx.upper_bound = controlFlux[i]

            sol_controlled = model_init.optimize(objective_sense=objType)

            objFlux.append(sol_controlled.f)

       # re-initialized by removing the constrains on controlRXN

        control_rx.lower_bound=-1000

        control_rx.upper_bound=1000

        fig = plt.figure(figsize=(8, 6), dpi=80)

        ax1 = fig.add_subplot(111)

        ax1.scatter(controlFlux, objFlux, linestyle='-')

        ax1.set_xlabel(controlRXn)

        ax1.set_ylabel('Objective: ' + obj_function)  # display the result with a scatter plot

        plt.savefig(controlRXn + '.pdf')

    else:

        print "The control reaction is not in the model, please double check"

    return None

#Function Test Code

methmodel=cobra.io.read_sbml_model("5GB1_WT.xml")

RXN=['EDD','ENO','ATPM']

robustnessanalysis(methmodel,RXN[2], 10, 'BIOMASS_M5GB1', 'maximize')

robustnessanalysis(methmodel,RXN[1], 10, 'BIOMASS_M5GB1', 'maximize')

robustnessanalysis(methmodel,RXN[0], 10, 'BIOMASS_M5GB1', 'maximize')

1. Flux ratio constraints

import cobra

import argparse

parser = argparse.ArgumentParser(description="Force fluxes of two reactions to fit a specified ratio")

parser.add_argument("Model", help="Model to be modified")

parser.add_argument("--reaction1", dest="r1", help="First reaction to be constrained")

parser.add_argument("--reaction2", dest="r2", help="Second reaction to be constrained")

parser.add_argument("--ratio1", dest="f1", help="Ratio value of first reaction")

parser.add_argument("--ratio2", dest="f2", help="Ratio value of second reaction")

parser.add_argument("-f", dest="format", default="xml", choices=["xml", "json", "mat"],

                                                                                help="Model format (xml, json, or mat, default=xml)")

parser.add_argument("-o", dest="outfile", default=None, help="Output model file (default = input model file)")

args = parser.parse_args()

outfile = ""

if args.outfile is not None:

                outfile = args.outfile

else:

                outfile = args.Model

model = ""

if args.format == "xml":

                model = cobra.io.read_sbml_model(args.Model)

elif args.format == "json":

                model = cobra.io.load_json_model(args.Model)

elif args.format == "mat":

                model = cobra.io.load_matlab_model(args.Model)

#Confirm specified reactions exist

try:

                reaction1 = model.reactions.get_by_id(args.r1)

except KeyError:

                print("Reaction {} not in model".format(args.r1))

                parser.print_help()

                exit()

try:

                reaction2 = model.reactions.get_by_id(args.r2)

except KeyError:

                print("Reaction {} not in model".format(args.r1))

                parser.print_help()

                exit()

#Create Ghost metabolite and add to reactions

ghost_id = "RBC_{}_{}".format(args.r1, args.r2)

ghost_metabolite = cobra.Metabolite(ghost_id, name=ghost_id, compartment='c')

reaction1.add_metabolites({ghost_metabolite: float(args.f2)})

reaction2.add_metabolites({ghost_metabolite: (-1 * float(args.f1))})

#Save constrained model

if args.format == "xml":

                cobra.io.write_sbml_model(model, outfile)

elif args.format == "json":

                cobra.io.save_json_model(model, outfile)

elif args.format == "mat":

                cobra.io.save_matlab_model(model, outfile)
